# Identification of a novel nonsense NOG mutation in a patient with stapes ankylosis and symphalangism spectrum disorder

**DOI:** 10.1038/s41439-023-00236-x

**Published:** 2023-04-13

**Authors:** Toru Sonoyama, Takashi Ishino, Yui Ogawa, Takashi Oda, Sachio Takeno

**Affiliations:** Department of Otorhinolaryngology, Head & Neck Surgery, Graduate School of Biomedical & Health Sciences, Hiroshima University, 1-2-3 Kasumi, Minami-ku, Hiroshima City, Hiroshima 734-8551 Japan

**Keywords:** Gene expression, Genetics

## Abstract

Multiple bone disorders due to mutations in the human noggin (NOG) causes a variety of phenotypes. Hearing impairment due to stapes ankylosis secondary to bony degeneration is also a feature of these syndromes. We describe the case of an individual in a Japanese family with conductive hearing loss due to stapes ankylosis and hyperopia and dactylosymphysis. We revealed a novel NOG mutation, NM_005450.6:c.222 C > A / p.Tyr74*, and confirmed genetic significance.

Stapes ankylosis is characterized by hearing loss due to congenital or acquired stapes fixation. While otosclerosis is the most common progressive conductive hearing loss caused by stapes ankylosis in adults, congenital stapes ankylosis is often difficult to differentiate from otosclerosis if diagnosed later in life. Congenital stapes ankylosis seems to be associated with osteodysplasty, such as osteogenesis imperfecta type I. It may occur as an isolated temporal bone anomaly, such as X-linked stapes fixation with perilymphatic gusher (DFN3)^1^. Additionally, some families reportedly have an autosomal-dominant form of congenital stapes ankylosis^1–3^. They describe clinical manifestations and corresponding bone abnormalities, including multiple synostosis, proximal symphalangism, and facial abnormalities. They also list several corresponding *NOG* gene mutations identified for these genealogy changes. Noggin is a secreted protein encoded by *NOG* that is essential for normal bone and joint formation in both humans and mice^4^, and appears to play a regulatory role as a bone morphogenetic protein (BMP) antagonist during joint formation. Mutations in *NOG* are in multiple autosomal-dominant syndromes that often share symptoms, such as conductive hearing loss, hyperopia, and digital anomalies^5,6^. These include stapes ankylosis syndromes such as proximal symphalangism (SYM1, OMIM 185800), multiple-synostoses syndrome (SYNS1, OMIM 186500), stapes ankylosis with broad thumbs and toes (SABTT, OMIM184460), tarsal-carpal coalition syndrome (TCC, OMIM 186570), and brachydactyly type B2 (BDB2, OMIM 611377). Currently, similarities and variations in overlapping features have led to the categorization of all these syndromes as *NOG*-related symphalangism spectrum disorder (*NOG*-SSD). Here, we describe the pedigree of a Japanese family with inherited stapes ankylosis, hyperopia, and digital anomalies, and show that a novel heterozygous *NOG* mutation results in *NOG*-SSD.

The proband (Case B: III-3) was a 27-year-old woman referred to our hospital due to bilateral hearing loss. She was not administered a newborn hearing screening test but seemed to have been aware of her hearing disability since early childhood and continued to suffer in her job due to hearing difficulties. Pure tone audiometry showed a bilateral conductive hearing loss, especially in the low pitch area. Although tympanometry yielded a type A curve, a stapedial reflex test indicated no response in either ear. A High-resolution computed tomography scan showed no ossicular malformation or inner ear abnormalities.

Her medical history and pedigree, including hearing loss, symphalangism, dactylosymphysis, brachydactyly, and hyperopia, were evaluated before surgery. Fig. 1 shows the pedigree of this family, which had five affected individuals. Physical findings and X-ray evaluation of her hands showed symphalangism and short, intermediate phalanges (brachydactyly) in two fingers (Fig. 2a, b, c). The range of motion in her elbow joint was restricted, and she could not touch her shoulders with her hands (Fig. 2d). An ophthalmologic examination revealed hyperopia. Two of her elder brothers (III-1 and III-2) also suffered from hearing loss, hyperopia, dactylosymphysis.

**Fig. 1:**
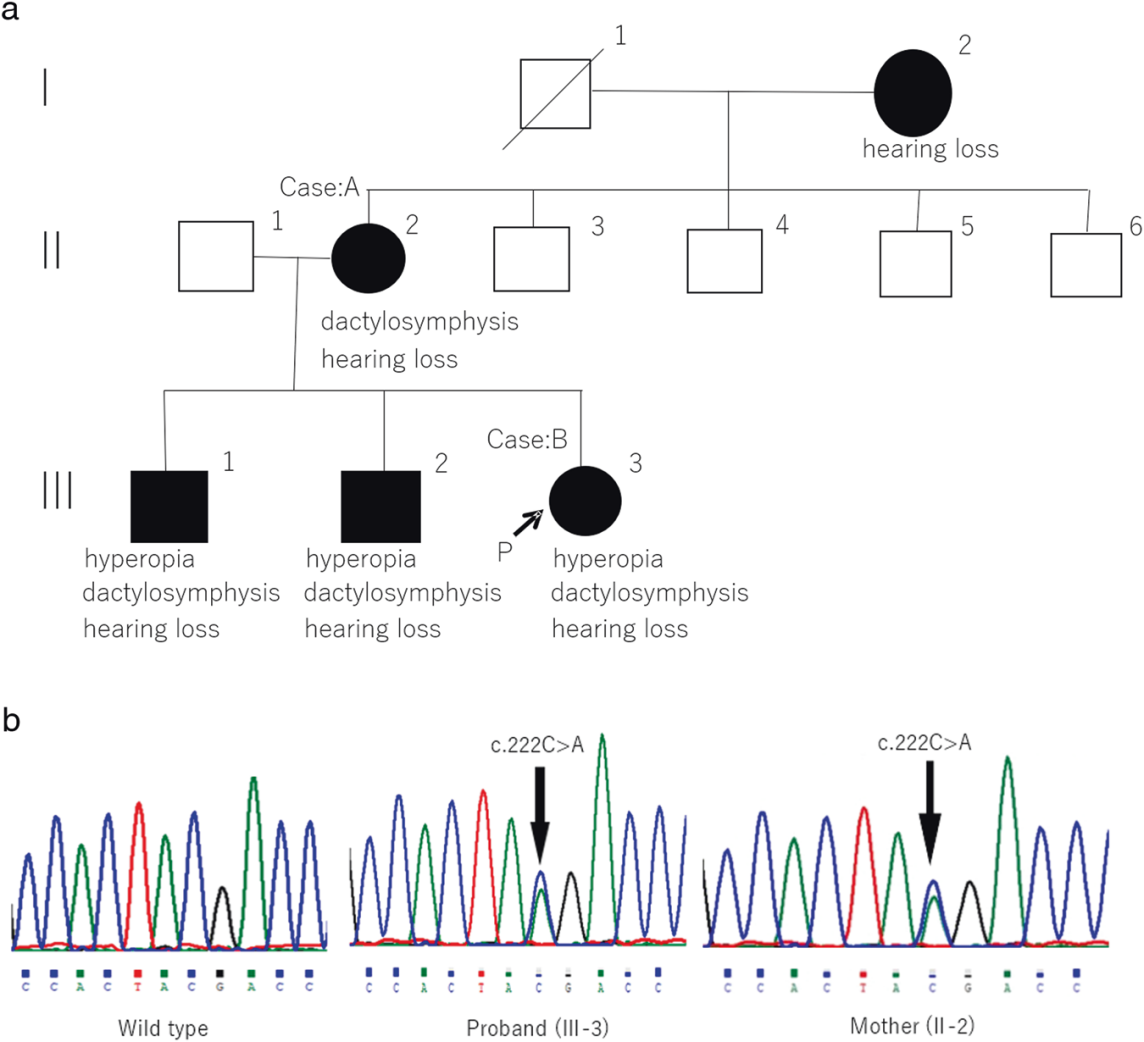
Pedigree and a variant of the NOG gene. **a** Family Pedigree. Square symbols denote male patients, and circles denote female patients. Blackened symbols denote affected individuals, and white symbols denote unaffected individuals. **b** Sanger sequencing chromatogram. Sanger DNA sequencing chromatogram demonstrates heterozygosity for a NOG variant (c.222 C > A / p.Tyr74*) in proband (III-3) and her mother (II-2).

**Fig. 2:**
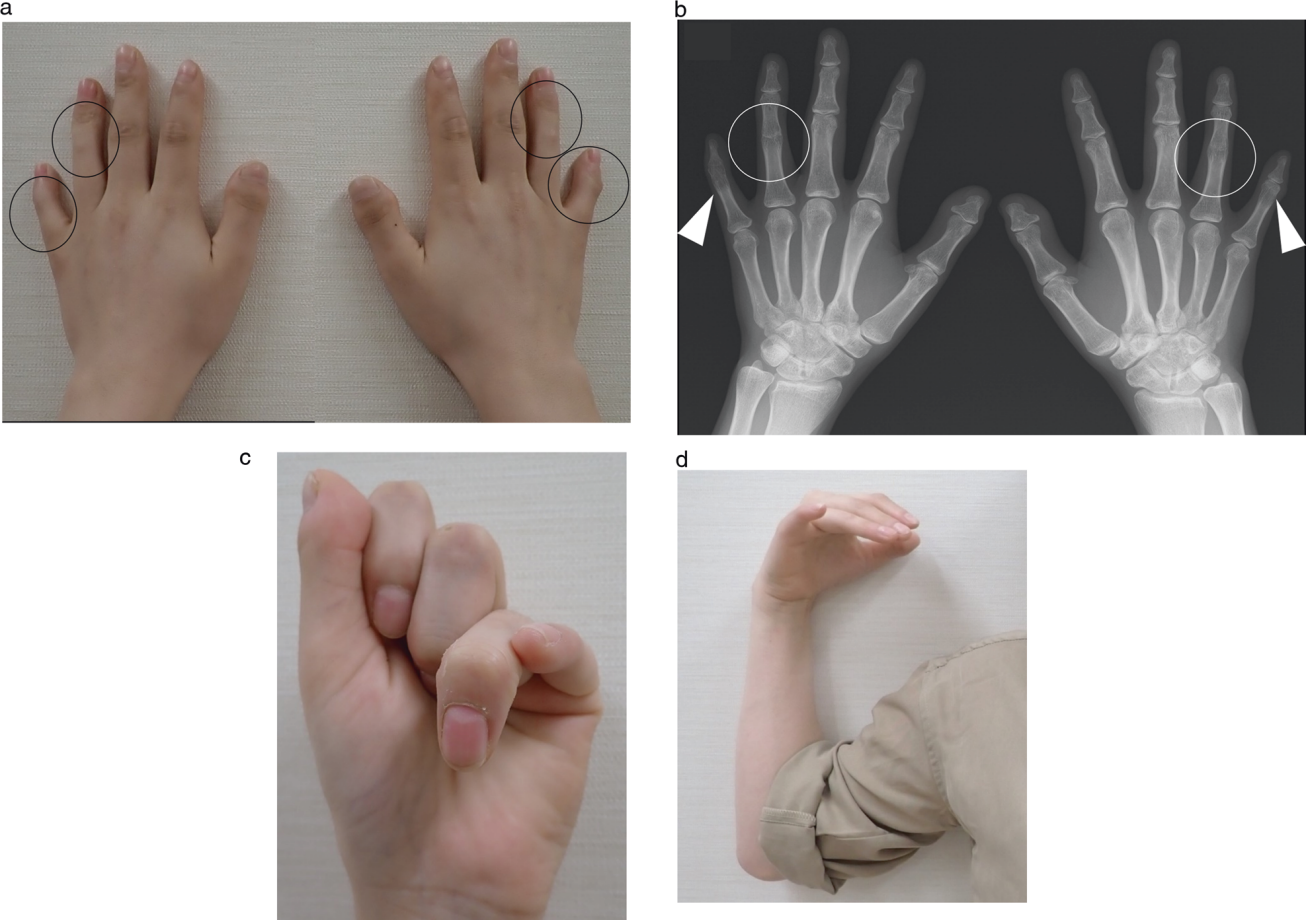
Phenotype of the patient. Phenotypic characteristics of the patient. **a** Photographs and **b** X-ray imaging of the hands of the proband. Circles indicate symphalangism, and arrowheads indicate brachydactyly. **c**, **d** Photograph illustrating a restricted range of elbow joint motion in affected individuals and their inability to touch their shoulders with their hands.

Blood samples were obtained from the proband (caseB: III-3) and her mother (caseA:II-2), and DNA was extracted from peripheral lymphocytes using the PAXgene Blood DNA kit (Qiagen#761133, Valencia, USA). As pedigree and clinical features were suggestive of mutations in *NOG*, we analyzed the NOG-coding region in each case by sequencing genomic DNA. Primer pairs were designed to include the entire protein-coding region consisting of 698 nucleotides, and the NOG-coding region was amplified by polymerase chain reaction using the following primers pairs: NOG (forward), 5'-CTCGGCGTGCTCTCCTC-3'; NOG (reverse), 5' - ATCGATCAAGTGTCCGGGTG-3'. DNA sequencing was performed using the standard protocol of the Applied Biosystems 3130 l gene sequence analyzer (Applied Biosystems, Nieuwerkerk aan de IJssel, The Netherlands). Study participants provided written informed consent. The Ethical Review Committee of the Hiroshima University, Japan, approved the research protocol.

Due to significant conductive hearing loss, we performed surgery on her left ear using an endoscope, which revealed ankylosis of the stapes footplate; hence, we performed a stapedotomy. The patient’s hearing threshold improved significantly, and her hearing level has remained stable for >1.5 years after surgery.

Genetic analysis identified a heterozygous c.222 C > A/p.Tyr74* mutation in *NOG* in the proband (Case B) and in her mother (Case A) (Fig. 1b), which has not been previously reported or cataloged in either the HGMD or other databases such as ClinVar. Given that other nonsense mutations such as p.Gln110* and p. Leu129* have been reported to be pathognomic, we hypothesize that this produces a truncated noggin protein that disrupts protein function.

The human *NOG* gene is present on chromosome 17q22, has one exon, and codes a secreted protein, Noggin, that seems to be involved in BMP signaling as an antagonist essential for bone formation in both humans and mice^4,7,8^. BMP promotes mesenchymal cell proliferation and differentiation into chondroblasts and osteoblasts and induces apoptosis during joint formation^9^.

NOG mutations have been previously described in the context of multiple bone diseases^10^, e.g., the typical features of SYM1 are narrow proximal interphalangeal joint space and symphalangism of the 4^th^ and/or 5^th^ fingers^11,12^. SYM1 is characterized by an autosomal-dominant heritability with minimal genetic heterogeneity, and mutations in *NOG* are thought to be predominantly responsible for SYM1; nevertheless, other genes such as GDF5 have also been implicated^13^. Thus, even though our patient presented with conductive hearing loss, a proximal phalangeal fusion of the fingers, and limited range of motion in the shoulder, we considered it reasonable to clinically diagnose her with SYM1 despite the absence of other systemic skeletal abnormalities or ocular symptoms. Additionally, a variety of syndromes, such as SYNS1, TCC, BDB2, SABTT, have been attributed to heterozygous *NOG*, and are described as subtypes of SSD.

Notably, the same variant of NOG can result in dissimilar phenotypes among different families or various affected members of the same family^14,15^. Gong et al. reported that the key features differentiating SYM1 from SYNS1 are characteristic physiognomy, hyperopia, and the absence of cervical vertebral fusion and symphalangism^16,17^. Similarly, it is hard to accurately categorize such patients as having a particular disease because, to date, >50 mutations in *NOG* are reportedly involved in a wide variety of bone development anomalies, as listed by Yuan et al^7^. Congruently, previous case reports confirm that mutations in the same *NOG*-coding sequence can lead to various phenotypes within one family^18–21^,indicating that disease expression may correlate to either location or type of *NOG* mutation.

We describe the pedigree of a Japanese family with SSD, and analysis of the genetic changes in the proband and her mother revealed a novel *NOG* c.222 C > A/p.Tyr74*. This mutation site is present between c.163 G > T/p.Asp55Tyr and c.328 C > T/p.Gln110*, which, according to Yuan et al.^7^, are pathognomic for SYM1 and FOP, respectively. Further, as these two mutations reportedly result in a termination codon and consequent degeneration of the noggin protein, a codon in this specific region is expected to be crucial for BMP7 binding; hence, its loss can affect joint morphogenesis. Congruently, evaluation of the pathogenicity of this mutation in “MutationTaster” and “Provean” confirmed its significance, as the results were “Prediction disease causing” and “Deteleous,” respectively. Thus, and under ACMG guidelines, we posit that the c.222 C > A mutation causes a meaningful change in *NOG* that is pathogenic (PVS1, PM2, PM3, and PP4).

The occurrence of bilateral congenital stapes ankylosis without other anomalies is rare, and it is sometimes not found in mild hearing loss. Further, congenital stapes ankylosis syndrome is difficult to distinguish from otosclerosis when first identified in adults. Thus, as some patients diagnosed with sporadic or non-syndromic familial otosclerosis may indeed have SSD, those with conductive hearing loss should be evaluated for other osseous lesions. The degree of hearing impairment in SSD is different from those described previously, i.e., mild to profound, possibly due to disease progression. Thus, attention must be paid to patients with suspected bilateral conductive hearing loss due to mutations in *NOG*. Unlike the NOG mutation reported in SYM 1 and SYNS1, family lineages with stapes ankylosis but no other fusions presumably have a protein with a dysfunctional terminal domain otherwise abundant in cysteine (C). Some pathogenic differences due to cysteine may exist among patients without osseous abnormality.

We identified a novel *NOG* mutation in a Japanese family, which helped clarify the relationship between phalangeal fusion disease and conductive hearing loss. Apart from genetic counseling, we could also provide beneficial information about progressive hearing loss, which would help affected siblings as they could recover hearing after surgery. Thus, managing patients presenting with bilateral conductive hearing loss requires careful physical evaluation of osteodystrophy based on both past and family medical histories.
