# Correction: Identification of a novel nonsense NOG mutation in a patient with stapes ankylosis and symphalangism spectrum disorder

**DOI:** 10.1038/s41439-023-00249-6

**Published:** 2023-11-15

**Authors:** Toru Sonoyama, Takashi Ishino, Yui Ogawa, Takashi Oda, Sachio Takeno

**Affiliations:** Department of Otorhinolaryngology, Head & Neck Surgery, Graduate School of Biomedical & Health Sciences, Hiroshima University, 1-2-3 Kasumi, Minami-ku, Hiroshima City, Hiroshima 734-8551 Japan

**Keywords:** Gene expression, Genetics

Correction to: *Human Genome Variation* 10.1038/s41439-023-00236-x, published online 13 April 2023

The original version of this article unfortunately did not contain the HGV database section.

## HGV Database

The relevant data from this Data Report was hosted at the Human Genome Variation Database at 10.6084/m9.figshare.hgv.3381.

